# A Resume of Treatment of Alveolar Abscess

**Published:** 1884-09

**Authors:** F. W. Sage

**Affiliations:** Cincinnati


### THE DENTAL REGISTER.

Vol. XXXVIII.] **SEPTEMBER, 1884.** [No. 9.

### Communications.

### A Resume of Treatment of Alveolar Abscess.

BY F. W. SAGE, D.D.S., CINCINNATI.

Read before the Mad River Valley Dental Society.

A well known contributor to dental literature speaks of the "always present abscess at or beyond the apex of a devitalized root." The fact, however, that dentists of unquestionable attainments,.who are supposed to know what they are about, not unfrequently cleanse and fill roots at a single sitting, without apparent apprehension of after trouble, would seem to indicate one of two things: either that they dissent from the opinion above quoted, or else that they are possessed of unusal powers of discrimination, as regards safe and hazardous cases which come into their hands. There is, however, no wonderful secret to be revealed regarding this off-hand and summary way of dispensing with the usual routine of treatment. It is doubtful whether those who occasionally adopt this course with dead teeth stop to make close and critical inquiry as to whether or not an abscess exists. They perhaps require no further reason by way of self-justification, than the fact that experience has taught them that, in certain cases and under certain circumstances, roots may at once be filled with impunity.

Reasoning *a posteriori*, we proceed to examine a case where a root has been thus filled without preliminary treatment for abscess, and with every appearance of success, as regards the non-occasioning of after-trouble. It is to be observed, in the first place, that this is a course of procedure recommended ten or a dozen years ago by Dr. Black, of Jacksonville, Illinois. It differs, however, essentially from Dr. Black's idea in that it is an attempt to evade the responsibility and trouble of treating for abscess, by avoiding a disturbance of the parts where abscess is suspected; whereas Dr. Black's deliberate purpose was, as we understood it at the time he promulgated the idea, to force the abscess presumed to be invariably present, to point on the gum, in other words, to convert the class of blind abscesses, so difficult to treat successfully through the root canal, into open abscesses, or abscesses with a fistula. While Dr. Black's idea was to proceed heroically, and regardless of the suffering entailed upon the patient, to reduce the abscess to a condition favorable for radical treatment, the treatment of which we now speak is based apparently in the idea of letting well enough alone and avoiding disturbance.

Dr. Black, it would seem, recognized the necessity of securing a counter-opening through the gum, as a requisite to successful treatment. His after-treatment of the case, as we remember it, was by the usual method of slitting up the fistula, securing a free opening through the alveolar walls, and stimulating the parts to healthy action. His method of procedure has been criticised on the ground that, while it involved much lingering pain and distress for the patient, it possessed no advantage over the generally approved method of lancing and drilling through the process in the first place, by way of forestalling nature's slower method of relieving the affected parts. It was probably a recognition of the wide difference sometimes existing between theory and practice which suggested the idea of the unheroic method we have referred to. The dentist discovered that the heroic method was quite sat, isfactory from his point of view; at the same time, perhaps, he discovered upon searching the records that he had never repeated for one and the same individual, the somewhat severe operation of lancing the gum, trephining the bone, and packing the cyst by means of a tent. He found that the radical course of treatment involved the infliction of considerable pain, and that the use of tents to keep the parts patulous and induce healing of the cyst by granulations from the bottom upward, sometimes meant severe inflammation, and two or three days' detention from business. He had observed that many dead teeth occasion no pain from month to month, and from year to year; and, as the result of patient experiment and many failures, he finally came to recognize certain cases which admitted of being at once filled.

This brings us back to the investigation of an imaginary instance to which we are triumphantly referred as illustrating the feasibility of dispensing with treatment. This tooth, it will be observed, is of solid, dense structure. We opine that the operator proceeded with extreme care and delicacy to remove the dead pulp, avoiding injudicious probing through the foramen. We venture the assertion that the pulp had not been so long dead that its consistency was entirely lost, and that if it was discovered under a broken or leaky filling, it was at least so protected that the presence of foreign particles had not caused it to become compacted in the canal. Everything points to the fact of the apical foramen having been found impervious to the finest nerve bristle. This conjecture, if it be correct, is significant; for it follows that in all probability no air found ingress through the foramen to excite active decomposition in the cyst, supposing there was a cyst. Probably the dentist gathered encouragement from the fact that, after having removed the decomposed pulp and washed out the canal repeatedly, there was no persistent, offensive odor such as he had noticed about old chronic cases. It was not one of those cases in which the structure of the root seemed permeated with putridity, so that nothing would sweeten it. Had it been such a case, he would have hesitated to do as he did, for he would have foreseen trouble almost certain to ensue.

Let us now consider and compare these different courses of procedure in filling roots, and award to each the merit to which it is entitled. When it is certainly known that a blind abscess exists at the end of a root, there is a certain advantage in lancing through the gum and trephining the bone, over the alternative of waiting for nature to establish a fistula. The advantage consists in this, that active inflammation thus artificially induced usually results more speedily in a cure than is possible, at least in old chronic cases where a fistula has long existed. Granting that it is possible in many cases, with proper precaution, to fill at once many roots which hitherto have been supposed to absolutely require repeated treatments before being filled, and assuming what is probably true, that the development of abscess is only a question of time, the long delay renders more and more difficult the final cure of the abscess. The wall of limiting fibrine, constituting the so-called pyogenic sac, gradually loses its vascular character, becomes thickened and indurated, and assumes an appearance, in time, of fatty degeneration as we often see about the roots of extracted teeth long diseased. This thickened membrane resists the action of escharotics, and indeed of any and all of the remedies commonly used, so that we find such old chronic cases practically incurable, excepting by means of an extensive operation with the knife, more popular in theory than in practice. Taken in their earlier stages, however, the vascular wall of limiting fibrine responds to the influence of tonics, astringents, or escharotics, and is converted into healthy tissue, or absorbed, or remains, at least, inert.

In the treatment of obstinate cases of long standing, the use of catgut, introduced through the fistula for the purpose of dilation, illustrates a principle in surgery the value of which is not fully appreciated by all dentists. The idea is, when stimulants and caustics have failed to secure healthy action, to break down the unhealthy granulations deep-seated in the cellular substance of the parts by continued pressure. This principle is applied in the treatment of leucorrhœa and of stricture of the urethra, and often effects cures when medication has been of no avail. A chronic fistula in the gum is simply an elongated abscess, which, by the use of a catgut tent, may be expanded sufficiently to excite healthy inflammation, eventuating in a cure without medicine.

But, says the advocate of no-immediate-treatment-for-abscess, let us present our claims. To begin with, we think you radical-cure men make altogether too much of this matter of curing abscesses. We are about prepared to say that we consider it of slight importance whether the abscess be *ever* cured, provided we can avoid irritating it when we fill the root. Do you not know that abscesses often remain for years encysted in other parts of the body without causing inconvenience? We claim that the condition of the parts is so ameliorated by the substitution, in the root or roots, of a good, solid, impervious filling, for the mass of putrifying matter removed, that the further treatment of the abscess is of little moment. Moreover, every time you pump medicine, or tepid water, or indeed anything through a root, so as to distend the sac, you run a risk of inducing pyæmia.

Very true, we admit, as to this last statement. Nothing of a fluid nature, not even tepid water, should be forced into a blind abscess, until after every effort has been made to drain off the accumulated pus. If a fistula exists, the danger is greatly diminished, because the fluid so introduced then simply drives out the pus through the opening. But to force carbolic acid especially, which, in a concentrated form is an irritant poison, into a blind abscess, is a most pernicious practice. It is doubtful whether its use beyond the apex of the root is ever indicated.

The true value of carbolic acid, as a destroyer of bacteria, is to be found in flooding the canal with this remedy during the operation of moving effete matter. A careful consideration of Lister's method of using carbolic acid to prevent pus-degeneration in treating wounds, suggests the possibility of at once treating and filling any root canal without danger of exciting severe inflammation. The canal should be kept constantly full by means of a drop tube. In two instances in which the experiment was tried by the writer, no more than a slight soreness followed. The dam was applied and the cavity flooded with carbolic acid from the instant attempt to enter the canal was made. The apex of the root was filled with lead driven in carefully, so as at the same time to force out the acid, afterwards the root was dried out and filled with gutta-percha.

Lister claims for this method of treating wounds, by rigidly excluding organic germs floating in the air that, "even in extensive lacerations and mutilations, union will take place without the formation of a single drop of pus, and without the occurrence of any general febrile reaction." This is disputed, however, by Ponchet, Bastian, and other scientists. Briefly stated, Lister's method consisted in protecting the parts with the vapor of carbolic acid, and with dressings of the same frequently renewed. In reading the details of his method of applying the acid, *frequently renewing* it, and never for an instant allowing the air to gain access to the wound, excepting after having been filtered through the medicated dressing, we may gain a hint about treating roots with this remedy. The query arises, is it sufficient to leave a few fibres of lint soaked with the medicament in the unsealed root, as is often done, with the idea of allowing drainage from the suspected abscess? We may also gather other hints from reading the details of his practice, as for instance, where he strictly enjoins constant care to protect the raw surface and edges of the wound from direct contact with the carbolic acid in the dressings, since it would infallibly produce sufficient irritation to cause suppuration. In another place he speaks of syringing out an incised or lacerated wound with a solution of carbolic acid, being extremely careful not to inject it forcibly so as to get up a high pressure, or the cellular tissue in the neighborhood becoming charged with the solution, will take on active inflammation.

Perhaps this will serve as a hint to those inquirers after light and information who write to the budget department of the journals, to know why a tooth which has enjoyed a life-long reputation for orderly walk and conversation, suddenly develops symptoms of total depravity under the well-intended ministrations of the dentist. The explanation suggests itself at once. One of two conditions probably existed about the root of that tooth ; there was either an old, chronic abscess filled with pus and partly disorganized matter, or a condition of the tissues favorable to inflammation, which is the precursor of abscess. The dentist used a broach which had already dried pus from somebody else's abscess upon it, or he used a clean broach (we won't insist upon complicating matters) and he pumped up carbolic acid, in full strength, into the root, and jobbed away at the foramen without succeeding, at first, in getting through; but at last he got through by a squeeze, and then he stopped, seeing no evidence of a fistulous opening. He wiped out the excess of acid, and thrust up a few fibres of silk floss, and told the patient to call the next day and let him see what progress it was making. The next day it was discovered that the progresss made was considerable, more indeed than either dentist or patient had anticipated. The patient said he had consulted his physician, in the night, and had been told he was in danger of blood-poisoning, and nothing the dentist could say, upon his word of honor, could convince that patient that he was not suffering from the effects of a half ounce or so of arsenic or strychnine pumped into his tooth. Or supposing it was not, after all, an old, chronic abscess, but only a young one, with vascular walls of limiting fibrine, and no sign of a fistula; the carbolic acid being pumped in through a minute orifice distended the parts, and penetrated the soft tissues, and severe inflammation was the result. The reason, probably, why severe inflammation is not so likely to follow when the foramen and the canal are large and easily penetrated, is either that the acid incidentally regurgitates, owing to the difficulty of making a cotton piston sufficiently dense to prevent leakage, or it may be, as in the case of an abscess of long standing, the walls of the sac, having become thickened and indurated, prevent the acid from escaping through into the healthy, inflammable tissue.

It will be noticed that when the pus in the sac can be easily induced to flow out through the root, by probing with a broach, the danger of inflammation is greatly reduced. In such a case, the pus being evacuated, the pernicious effect of germ organisms allowed ingress to the sac is but little to be feared; and the very fact of pus being found where there is no indication of recent inflammation, indicates a wall of limiting fibrine, which may not easily be provoked to inflammation by injecting carbolic acid, though it might be so affected by using a strong solution of chloride of zinc.

But, some one inquires, admitting the truth of all this, what is the remedy? You profess to have explained the manner and cause of the mischief; how will you treat a blind abscess so as to avoid all this? In reply, we refer the questioner to Dr. Flagg's prescription: lance through the gum, and drill through the bone to the apex of the root threatened with abscess, and so anticipate trouble. It is our private opinion that this is rather heroic treatment, and in actual practice we have found few who would more than once submit to it. Moreover, it is not always possible to strike the sought-for apex; and, finally, the abscess is not always directly at the apex. There are two other methods of treatment which are not without merit. The first is to wash out the canal with a solution of carbolic acid, one part in thirty-three parts of distilled water, avoiding distension of the sac as much as possible, and coaxing a flow of matter if possible. We do not favor drilling out the apex ordinarily. Every dental surgeon must have had occasion to notice that a canal, apparently impermeable, may be easily entered on the second or third day, or after the pus has begun to flow. This solution of carbolic acid will be far less likely to occasion inflammation, even though it penetrate the cellular tissues, than the acid in full strength, which, when concentrated and confined, is an irritant poison. The solution is sufficiently strong to kill bacteria. The after treatment of the abscess may be completed with a solution of sulphate of zinc, one grain to the ounce of distilled water. This supplements the treatment with the carbolic acid solution, since the sulphate of zinc is stimulating, and is a better astringent than carbolic acid, and does not, like chloride of zinc, destroy tissue, but simply hardens it, and favors the closing upon themselves of the walls of the now emptied abscess.

The query arises, by the way, whether the use of powerful corrosives injected into a hidden abscess is a rational course of treatment? What object is sought to be obtained? We can conceive of the necessity of a caustic application upon an exposed ulcer, from which the resulting eschar may be lifted away at the proper time, but what becomes of the wall of limiting fibrine in a circumscribed abscess, after having been burned with a caustic? It either forms a horny mass, or is converted into granular matter, or calcifies, and the conclusion would seem to be that relief by an operation with the knife, to remove the foreign particles, becomes necessary, after all. Why not then resort to the knife in the first place; break up the sac and remove it, leaving a healthy surface favorable for granulations.

Now, the idea of dispensing as far as possible, with any treatment for abscess, filling the root at once, if that be considered possible, is, we strongly suspect, based on a growing belief that an abscess, silently and slowly developing at the end of a filled root, is no very serious matter at the worst. It is seen to remain there, month after month, and even year after year, in many cases, occasioning little or no inconvenience. It is remarked that even after the most thorough and heroic treatment, abscesses recur in not a few cases. "If I can manage to do it without raising a disturbance, I propose to fill this root at once, and take my chances of having to treat the abscess through a fistula, later on," says the operator. "I am not sure this patient will return to have it treated now, if I set about to cure the abscess; I will fill the tooth and get my fee, and be done with it at once. If I should set out to treat this abscess, the chances are he would come in to-morrow with a swelled face, and call me a pirate; whereas, if he comes in a year hence, with one eye closed and his lip puffed out like a turnover, I can lecture him on the evils of high living, and reckless exposure; and he won't think I ought to apologize and make him a present of five dollars for making his tooth ache."

If we knew more about the subject of diathesis, we might avoid much feeling-of-our-way to avoid trouble. What are the signs of the inflammatory diathesis? Nobody knows very definitely. We know, however, that our red-haired, freckled-face, florid-complexioned patients require careful handling; so also does the individual with the bulbous nose, whose visage betrays bibulous excesses; so also does this pale-faced, hollow-eyed young Miss, whose nervous system has been over-stimulated in the school-room. Persons of a full habit induced by over-feeding, those of a gouty or rheumatic turn, as well as those suffering from malarial poison, usually exhibit the inflammatory diathesis. Scrofulous subjects offer but little encouragement to the dentist who would illustrate the certainty and ease with which an alveolar abscess may be cured. The scrofulous diathesis may be recognized by the following physical characteristics, as defined by a well-known authortiy: "There are two classes of individuals who exhibit this diathesis—the first have dark skin; large, prominent, black eyes; high cheek bones; wide, dilated nostrils; thinly chiseled lips, limbs disproportionately long, feet large, and instep low. They usually have a lounging, or sidling gait, and are, in many instances, knock-kneed, their toes having a tendency to turn in. Persons of this description are peculiarly susceptible to malignant disorders, resulting from inflammation. In such, the ravages of syphilis are terribly exhibited, if the disease be contracted.

The second class of scrofulous subjects presents quite a different physical aspect. The skin is light, fine, and soft; hair red, flaxen, or at least sandy or brown; eyes light or dark blue; heads large, and nearly always unduly developed in the intellectual regions; eyes prominent and marked by a dreamy look, or at times by great vivacity of expression. Their necks are too long and slender for their heads; their breasts are flat, and chests narrow, and they have, so to speak, no back bone. Individuals of this class are disposed to take on diseases which find a lodgment in the mucous structure." So much for diathesis.

A species of abscess, not very readily cured, in which it seems impossible to stimulate healthy action, is not unfrequently noticed by the dentist. It does not seem to be associated, necessarily, with any affection of the bone: is usually easily accessible to the introduction of medicine; is indolent, and discharges, on pressure, a thin, sanious, or ichorous pus. Liquids may be injected in unusual quantities through the root-canal, before presenting an appearance at fistulous vent; and the impression grows upon the dentist that they are widely diffused through the tissues, and not simply received into a reservoir or sac. This class of abscess probably belongs to what is described in general as the *diffuse* abscess, having no wall of limiting fibrine, and is a common sequel to pyæmia.

In conclusion, let us consider, very briefly, the relative value of some of the remedies in common use for treating abscess. Carbolic acid, properly used, is valuable for its antiseptic, as well as for its anæsthetic and stimulating properties. Salicylic acid is a better antiseptic, and is free from odor, but is not, like carbolic acid, volatile in its nature, and for that reason is considered inferior to the former acid, by those who regard the vapor as efficient in preventing decomposition. In recent abscess, it is not less irritating than the former, and is, perhaps, to be regarded as possessing few if any real advantages over carbolic acid.

Tincture of iodine is a valuable agent, on account of its stimulating power, and its peculiar influence in relieving congestion, by relieving the engorged capillaries. Used in full strength, it is too active an agent to inject into a recent abscess; but diluted with an equal quantity of alcohol, it may be safely used.

Peroxide of hydrogen seems to be a powerful cleansing and stimulating agent, and from reports of those who have used it extensively, it appears that it is, least of all remedies in use, liable to excite inflammation. It is doubtful whether its use alone will eventuate in a cure of alveolar abscess, but as an agent to drive out most effectually the pus in the sac, it may safely be relied on.

Eucalyptus oil has apparently effected cures; but its powers, as an antiseptic, stimulant, astringent, or what not, are not definitely and unequivocally rated in the dispensatories.

Boracic acid has antiseptic powers inferior to either carbolic or salicylic acid; but it is proportionately less irritating to a raw surface.

It is stated as a fact worthy of the attention of dentists, that a few drops of glycerine in a vial of carbolic acid, greatly increase the facility of ingress through apparently closed foramina. Experiment has shown that while the pure acid could not be forced, under heavy pressure with a piston, to penetrate an exceedingly minute opening in a steel plate, the object was readily accomplished by adding three or four drops of glycerine to an ounce of the acid.

